# Evaluating the Family Nurse Partnership Programme in Scotland: a natural experiment using linked data from health, education and social care.

**DOI:** 10.23889/ijpds.v7i3.1867

**Published:** 2022-08-25

**Authors:** Rebecca Cannings-John, Michael Robling, Fiona Lugg-Widger, James White, Julia Sanders, Mandy Lau

**Affiliations:** 1 Cardiff University

## Objectives

The Family Nurse Partnership (FNP) is an intensive home visiting service for teenage ﬁrst-time mothers, developed/trialled in the US and adapted/trialled across Europe. The Scottish Government aims to build on and supplement the existing evidence base for FNP, to assess eﬀectiveness of the programme, for future improvement work in Scotland.

## Approach

The commissioned natural experiment of FNP, takes advantage of existing information infrastructures in Scotland from health, education and social care and FNP programme implementation data. A cohort design was used covering ten Health Boards in Scotland. The cohort included first-time teenage mothers enrolled as FNP Clients between 2010 and 2016 and women who met FNP eligibility criteria but were pregnant when the programme was not recruiting (Controls). Outcomes are mapped to the Scottish FNP logic model and include those being tested statistically (n=34) or, where direction of effect is uncertain, outcomes are rare or data quality poor, descriptively (n=20).

## Results

We established a model of data linkage to routine Scottish data. Approvals were obtained to access data on over 8000 mothers (FNP Clients and Controls) and their children which formed the study cohort and were mapped to routine health, education and social care data. Outcomes covered maternal (health (smoking, alcohol/drugs), subsequent pregnancies/births, education) and child (health, development, education, protection) domains. Observed baseline imbalances between study arms will be described and adjusted for in modelling. Results will be published by the time of the conference and include sensitivity analyses that explore a priori sub-groups, variation by health board, year of booking and programme dosage.

## Conclusion and Relevance

The impact of this evaluation will establish a robustly matched study cohort, develop the evidence base on FNP in Scotland and internationally, develop a more streamlined linkage and approval process, and identify outcomes for short-term follow-up and importantly for a longer-term follow-up of the mothers and their children.

